# Annotations

**Published:** 1911-06-24

**Authors:** 


### ANNOTATIONS.

### A Medical Strike in Austria.

Much dissatisfaction is expressed among the profession in Austria at the new legislative proposals made by the Government with regard to "social reform." The doctors believe that if these proposals become law they will be absolutely ruined. As they have made up their minds to strike rather than submit, their Association has met and drawn up a moderate tariff so as to allow all citizens, including the poorest, to receive treatment during the strike on condition that the fee is paid on the spot. Families whose income is below 1,200 crowns will pay a crown, or a little over tenpence, a visit. If the doctor calls at the patient's house, or has to make a lengthy journey, or if the income of the family exceeds 1,200 crowns, the tariff is raised appreciably, though it is still moderate. Consultations will cost from five to ten crowns. The Association has apprised the public of its decision to strike, and of its scale of fees, at the same time explaining that the attitude taken up by the doctors is the result of the proposed law, which would render private medical practice impossible in poor and middle-class families.

### National Health Promotion Scheme.

The National League for Physical Education and Improvement is promoting a scheme for commemorating King Edward VII. and the interest he constantly took in all questions of the health and happiness of his people. The proposal put forward is to erect a popular Museum of National Health, readily available for the masses in London, and for visitors to the metropolis. As a central and convenient site the vacant land in Kingsway is suggested. The museum should accommodate a permanent collection and should also furnish duplicate material for the equipment of travelling vans and lecturers. It might also become a loan centre for the distribution of replicas of its models and diagrams for circulation among schools and institutions, and should be a model in constant close relation with the provincial museums, to which it would probably, in the opinion of the League, give birth. Further, the building should form the nucleus for the co-ordination of the various health-promoting institutions, both metropolitan and national; a well-equipped library is regarded as a vital and integral portion of the museum. Another suggestion is that the British pavilion of the International Hygiene Exhibition at Dresden this year should be retained and handed over to the authorities of the projected museum. The financial and general administration would best be framed on the lines of the King's Hospital Fund, with a separate department of the staff for the technical working of the institution. All courses of instruction, etc., would be provided at very low fees, so as to render the facilities of the museum available to all classes; and an appeal is to be made for £5,000 a year in subscriptions as well as for £100,000, the estimated cost of building and equipping the museum.

### The Marie Feodorovna Prizes.

At the International Conference of the Red Cross Society, which is to be held at Washington in May 1912, the Marie Feodorovna prizes will be awarded. These prizes are obtained from the interest accruing from a fund of 100,000 roubles presented some ten years ago by the Dowager-Empress of Russia for the purpose of diminishing the sufferings of the sick and wounded in times of war. The prizes for 1912 will consist of one of 6,000 roubles, two of 3,000 roubles each, and six of 1,000 roubles each. The subjects set for the competition are: (1) Organisation of evacuation methods for wounded on the battlefield, involving as much economy as possible in bearers. (2) Surgeon's portable lavatories for war. (3) Methods of applying dressings at aid stations and in ambulances. (4) Wheeled stretchers. (5) Support for a stretcher on the back of a mule. (6) Easily portable folding stretcher. (7) Transport of wounded between men-of-war and hospital vessels and the coast. (8) The best method of heating railroad cars by a system independent of steam from the locomotive. (9) The best model of a portable Röntgen-ray apparatus, permitting utilisation of $x$-rays on the battlefield and at first-aid stations. The largest prize will be awarded for the best solution of any question irrespective of what the question may be. Information can be obtained from the Exhibit Committee, Red Cross Society, Washington, D.C.

### Cockles and Mussels.*

Dr. Bulstrode's report on shell-fish other than oysters in relation to disease is a supplement to the one he made in 1896 to the Local Government Board with regard to "Oyster culture in relation to disease." As the result of the present inquiry it appears that cockles and mussels can only be consumed with comparative safety if they are cooked immediately before consumption. The methods in vogue for laying down, washing, and storing these shellfish are so unsatisfactory, and so little care is exercised in the choice of places from which they are collected, that it is courting disaster to consume them in a raw condition. At present practically no control is exercised by the sanitary authorities over the beds where the shellfish are found or over the manipulations to which they are subjected prior to being placed on the market. It is to be hoped that in consequence of this report public interest will be sufficiently aroused as to induce some definite action being taken by the authorities to ensure the defects being remedied. In the event of no such steps being taken, the public will have to protect itself by abstaining from such shellfish, a course which would mean the ruin of an industry and the loss of a foodstuff which under proper conditions of storage, collection, and washing should be wholesome and nutritious.

* Thirty-ninth Annual Report of the Local Government Board, 1909-10: Report on Shellfish other than Oysters in Relation to Disease. By H. Timbrell Bulstrode, M.D., with an introduction by the Medical Officer of the Board. (London: Darling and Sons, Ltd. Pp. 243, with 33 maps. 1911. Price 8s.)

